# Vegetable proteins as encapsulating agents: Recent updates and future perspectives

**DOI:** 10.1002/fsn3.3234

**Published:** 2023-01-27

**Authors:** Fakhar Islam, Yuosra Amer Ali, Ali Imran, Muhammad Afzaal, Syeda Mahvish Zahra, Maleeha Fatima, Farhan Saeed, Ifrah Usman, Umber Shehzadi, Shilpa Mehta, Mohd Asif Shah

**Affiliations:** ^1^ Department of Food Sciences Government College University Faisalabad Pakistan; ^2^ Department of Food Sciences, College of Agriculture and Forestry University of Mosul Mosul Iraq; ^3^ Department of Environmental Design, Health and Nutritional Sciences Allama Iqbal Open University Islamabad Pakistan; ^4^ Institute of Food Science and Nutrition, University of Sargodha Sargodha Pakistan; ^5^ Department of Home Economics Government College University Faisalabad Pakistan; ^6^ Department of Electrical and Electronic Engineering Auckland University of Technology Auckland New Zealand; ^7^ Adjunct Faculty University Center for Research & Development, Chandigarh University Mohali India

**Keywords:** active, coacervation, core, encapsulation, spray drying, vegetable proteins

## Abstract

The use of proteinaceous material is desired as it forms a protective gelation around the active core, making it safe through temperature, pH, and O_2_ in the stomach and intestinal environment. During the boom of functional food utilization in this era of advancement in drug delivery systems, there is a dire need to find more protein sources that could be explored for the potential of being used as encapsulation materials, especially vegetable proteins. This review covers certain examples which need to be explored to form an encapsulation coating material, including soybeans (conglycinin and glycinin), peas (vicilin and convicilin), sunflower (helianthins and albumins), legumes (glutenins and albumins), and proteins from oats, rice, and wheat. This review covers recent interventions exploring the mentioned vegetable protein encapsulation and imminent projections in the shifting paradigm from conventional process to environmentally friendly green process technologies and the sensitivity of methods used for encapsulation. Vegetable proteins are easily biodegradable and so are the procedures of spray drying and coacervation, which have been discussed to prepare the desired encapsulated functional food. Coacervation processes are yet more promising in the case of particle size formation ranging from nano to several hundred microns. The present review emphasizes the significance of using vegetable proteins as capsule material, as well as the specificity of encapsulation methods in relation to vegetable protein sensitivity and the purpose of encapsulation accompanying recent interventions.

## Graphical abstract

During the boom of functional food utilization in this era of advancement in drug delivery systems, there is a dire need to find more protein sources that could be explored for the potential of being used as encapsulation materials, especially vegetable proteins. This review covers certain examples which need to be explored to form an encapsulation coating material, including soy beans (conglycin and glycinin), peas (vicilin, convicilin), sunflower (helianthinins, albumins), legumes (glutenins, albumins), and proteins from oats, rice and wheat.

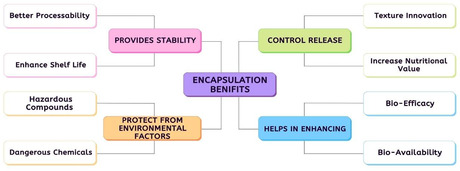

## 1 INTRODUCTION

Functional foods fortified with bioactive components are gaining attention worldwide. Despite these rising trends, the usage of free bioactive components in a variety of food as well as nutraceutical items can have a deleterious influence not only on the quality features of the product but also on its nutritional content (Gharibzahedi & Smith, 2021a, 2021b). Unpleasant taste, flavor, poor stability, and bioavailability are all examples of these undesirable changes. Encapsulation technology is critical in this context for overcoming these technological difficulties (Jafari, 2017). This approach is extremely demanding since it provides unique delivery systems for many bioactive components with increased chemical stability, bioavailability, digestibility, and solubility (Quintero et al., 2018). Microencapsulation is a popular method for stabilizing and protecting various vulnerable components from the bad influence of the environment. This technology has been shown to be a successful replacement in a variety of industries over the last several years since it maintains the appropriate effectiveness of additives and regulates optimal medication release and dose (Jarpa‐Parra, 2018). Researchers are paying more attention to naturally derived chemicals now that they have been labeled as generally recognized as safe. Numerous polysaccharides, including starches, chitosan, gums, pectin, and maltodextrins, have been used as encapsulating agents, among other substances (Carbonaro et al., 2015). Proteins are a diverse collection of molecules with particular structures, biodegradability, self‐association ability, biocompatibility, and amphiphilic character. Improved emulsifying, foaming, and gelling qualities are among the other techno functional attributes. Other unique plant protein activities are particularly encouraging since these qualities have provided an elevated ability for encapsulation of such proteins, which has been intensively explored in recent times (Gharibzahedi & Smith, 2020). These excellent properties include biodegradability, ease of availability, and improved physicochemical properties which are economically advantageous and may be employed as a preferred coating agent. In contrast to this, their production requires the use of few natural sources (Sharif et al., 2018). Microencapsulation is the separation of active ingredients (in aqueous, solid, or gaseous form) to generate products of circular shape and micrometer dimension, where the active ingredient as well as the core are insulated from the external atmosphere by a barrier (Quintero et al., 2017). This method could be used for a variety of reasons, including the protection of susceptible ingredients from their atmosphere, the advancement of regulated discharge characteristics, the disguising of undesirable tastes and odors of the ingredients, the dispersion of basic content when it should be utilized in extremely small quantities, and the transition of fluid substances into the mobile solid materials. Encapsulated components can be made via a variety of techniques, including interfacial polymerization, emulsion polymerization, solvent‐based evaporation, gel‐based formation, supercritical fluid expansion, coacervation/phase, fluidized bed, spray‐drying, and extrusion (Dubey et al., 2009). The microencapsulation approach used for a specific process will be determined by the dimension, biocompatibility, and degradability of the tiny particles required, the physiochemical characteristics of the center and covering, the administration of the microscopic particles, the suggested method for effective core discharge, and also the production expenses (Favaro‐Trindade et al., 2010). Soybean protein isolates, sunflower protein, oat protein, rice protein, legume protein, pea protein isolate, and wheat cereal protein are the most common vegetable proteins utilized as cladding in microencapsulation. Soy proteins offer functional qualities that make them excellent for encapsulation, like foaming, fat and water absorption, gelation, emulsion stability, and solubility, as well as superior organoleptic and film‐forming capabilities (Franzen & Kinsella, 1976). Cereal proteins like wheat, barley, oats, and corn are quite nutritionally beneficial, so they have received studies and commercial interest as a result. This review discusses recent research on using plant protein in microencapsulation. The impact of protein active characteristics and the microencapsulation technology on procedure effectiveness and microparticle characteristics is examined in detail.

## 2 ENCAPSULATION

**FIGURE 1:**
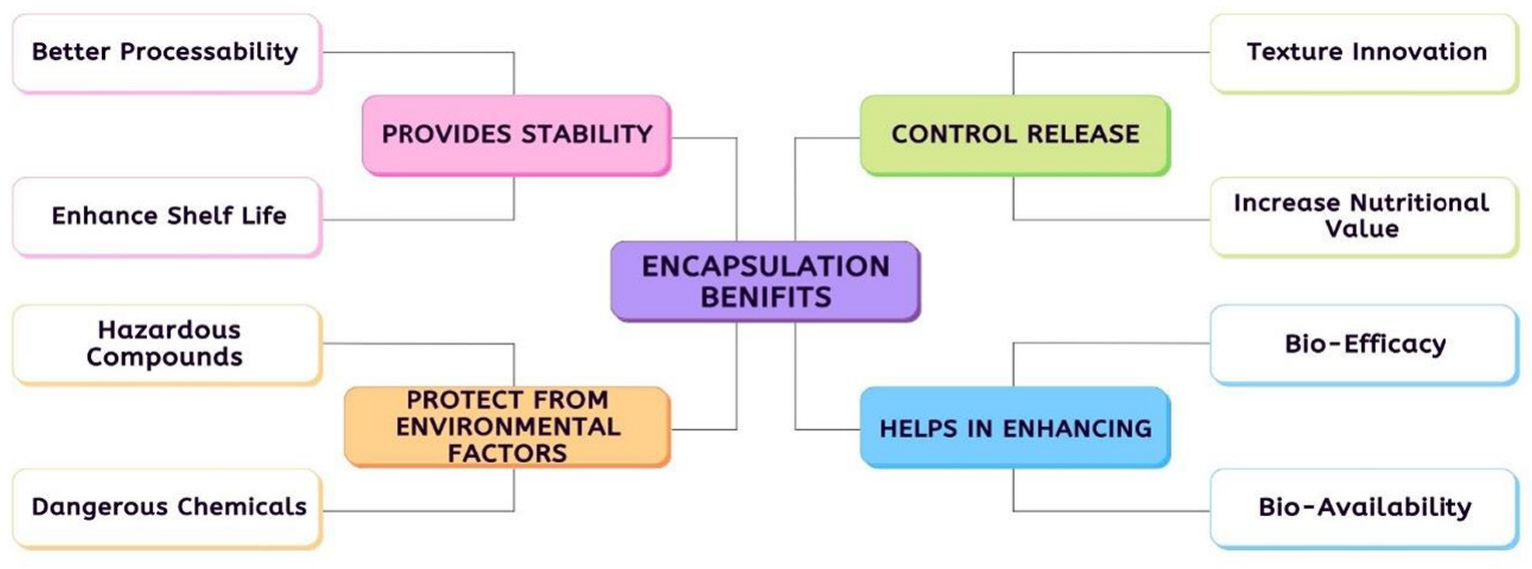
Flow sheet depicts the benefits of encapsulation

The process of encapsulation is identified as a mechanical/physiochemical approach to shield and segregate sensitive components, mainly of a bioactive nature, from atmospheric conditions, i.e., pH, O_2_, light, and aspects, that affect the activity of bioactive composites adversely. Bioactive composites can be present in any form of matter. The main material protecting these sensitive composites are covering/coating materials; polymer in nature that prevent and manage the timely availability and play a role in protection of its stability (Rocha et al., 2012). The encapsulation approach has a benefit in contrast to the immobilization method since it assures that the active components are completely enclosed by the use of coating material. Few pieces may be left exposed to the outside environment when using the immobilization approach as opposed to the encapsulating process. The encapsulation approach provides a benefit over the immobilized technique since it assures that the active components are completely enclosed by the use of coating material**.** Few pieces may be left exposed to the outside environment when using the immobilization approach as opposed to the encapsulating process (Papalamprou et al., 2005). Materials that have been enclosed are split into two major types, namely nano and micro, based on their sizes. Nanoparticles have a size range of 20–500 nm while microparticles have a size range of 1–200 microns. The lamination enclosing the compound is well‐known to the common population with different names, i.e., wall capsule, shell, cover, matrix, carrier, membrane, and encapsulate. The composite/substance that is covered within it is called an internal, active, core, or fill (Gharsallaoui et al., 2010); Figure 1.

## 3 ENCAPSULATION STRATEGIES FOR VEGETABLE PROTEINS

Coacervation and spray‐drying are the main methods used to microencapsulate the active ingredient by vegetable proteins. Vegetable proteins are resources that are renewable and biodegradable, and both procedures are “green” in that they do not require organic solvents utilization; procedures including solvent evaporation and gelation methods can also be taken into consideration (Dubey et al., 2009). A continuous process called spray‐drying turns from a liquid starting point to a rigid powder of tiny particles. It is a highly popular method of dehydration utilized to create a continuous matrix around the active ingredients. A stream of hot air is sprayed into the suspension/initial liquid/solution/emulsion, holding the core and wall ingredients. Instantaneous powder manufacturing is achieved by evaporating the solvent, which is almost often water (Nesterenko et al., 2013). This technology has many benefits, including ease of use, affordability, speed, and widespread industrial use. Readily soluble nature of cover substance in aqueous (or other specified solvent) and its reduced viscous nature at high solid concentration are crucial for efficient microencapsulation through spray‐drying. The disadvantages of this method include the potential for sensitive products to degrade at high drying temperatures as well as the loss of a sizeable amount of product (caused by the microparticles' adherence to the spray‐dryer wall). By precipitating wall‐forming elements around the active core in response to a variation in temperature or pH, or the inclusion of an electrolyte or nonsolvent component, microencapsulation by coacervation can be accomplished. The creation of a polymeric network surrounding the core is the result of this regulated desolvation. A chemical or enzymatic cross‐linker can solidify this layer of coacervates. Coacervation can happen in two ways: simply or intricately. Simple coacervation produces a single polymer envelope from the production of a single colloidal solute. Mixing two oppositely charged polyelectrolytes results in complex coacervation, which forms a shell covering a functional core. The sensitivity of coacervates to pH and ionic strength is another aspect that restricts their application in encapsulation (Wilson et al., 2007). The stability of proteins in acid medium is a crucial factor to take into account when using them in encapsulation systems. Because of their isoelectric point, proteins are vulnerable to precipitation and insoluble in the majority of acidic environments, particularly when core materials of acid nature are employed (i.e., vitamin C/ascorbic acid). The isoelectric point for the vegetable proteins’ bulk in water‐based solution lies in the pH range 3–5. Because of this, these biomaterials are typically utilized in alkaline environments to get strong protein solubility and effectively encapsulate active ingredients (Augustin et al., 2006). Depending on the technique selected, particle parameters like shape, size, and releasing characteristics might vary greatly. Microcapsules and microspheres are the two types that are primarily produced using the spray‐drying and coacervation process. While the dimension of particles produced by the coacervation system can range from nm in size to hundreds of microns, the spray‐drying procedure normally produces particles between 1 and 50 microns (Hu & Gan, 2008). Both these methods give high‐value microencapsulation efficiency (MEE), up to 100%.

## 4 ENCAPSULATED VEGETABLE PROTEINS

### 4.1 Soybean proteins

**FIGURE 2:**
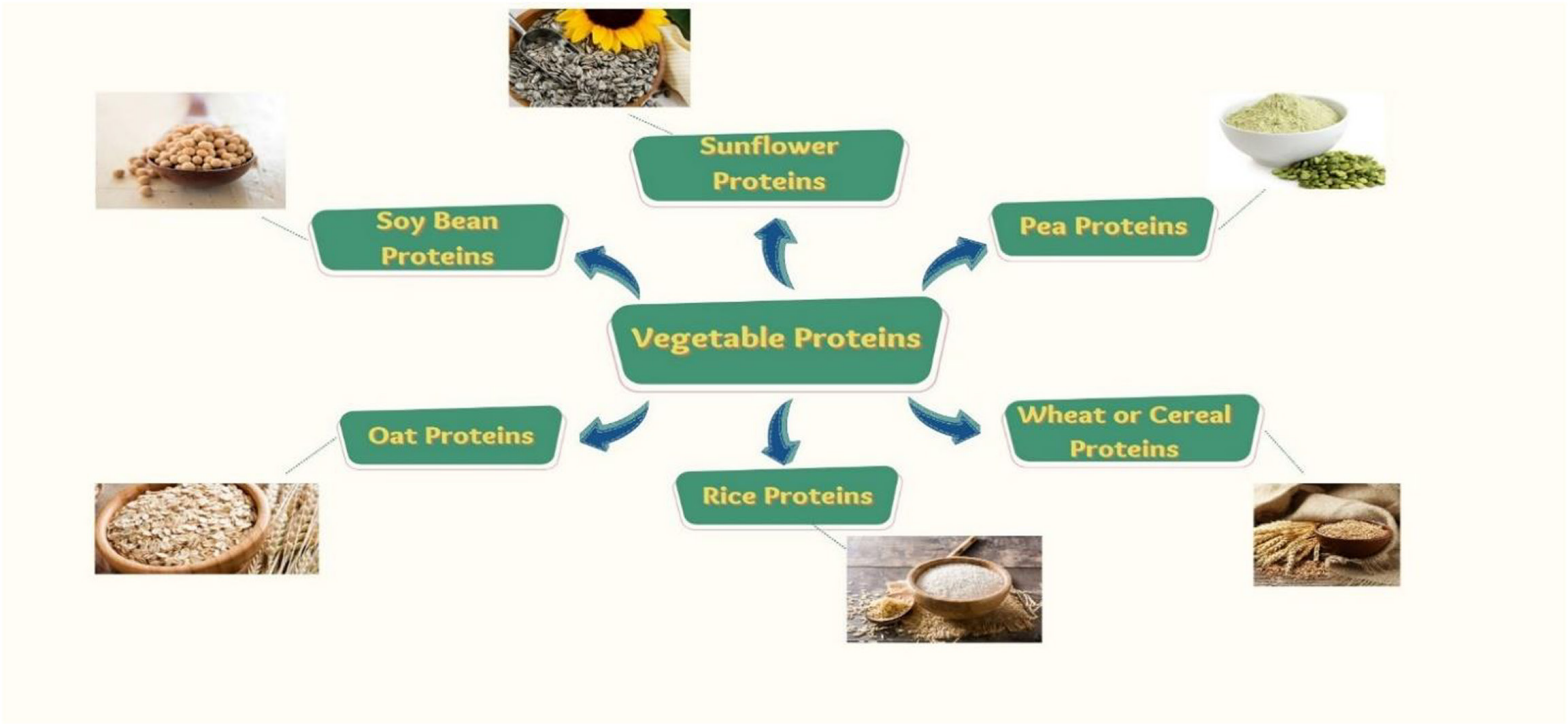
depicts the encapsulated proteins

The main proteins which are present in seeds of soybean are conglycinin and glycinin, which carried out a high fraction of about 35%–40% of total protein **(**Figure 2). The molecular weight of glycinin and conglycinin is about 350 kDa and 70 kDa, respectively. Interesting physicochemical and functional characteristics, including mucilaginous, emulsification, and detergent capabilities, may be seen in isolated and purified soy proteins (Bales et al., 2013). The heat treatment, pH, salts, and other substances' concentration and even presence have a significant impact on these protein properties as well as their solubility (oil, carbohydrate, and surfactant). Several writers have already researched the use of soy protein isolate (SPI) in microencapsulation. SPI can be combined with polysaccharides and utilized as a stand alone coating agent (Rusli et al., 2006). Better defense, oxidative stability, and drying qualities are favored when proteins and carbohydrates are used as a carrier material (Jiang et al., 2007). Spray‐drying is the primary method for creating microparticles because of SPI hydrosolubility, but coacervation and gelation have also been studied (Gan et al., 2008). A water‐in‐oil emulsion is created prior to the encapsulation stage in the case of hydrophobic core microencapsulation (Nesterenko et al., 2012). Due to the intriguing outcomes in terms of emulsion characteristics and stability, high‐pressure homogenization is frequently used to create these emulsions (Gharsallaoui et al., 2007). The size of the oil droplets and the viscosity of the emulsion both slightly decrease as the homogenization pressure is increased (Yu et al., 2007). A modest increase in microencapsulation effectiveness was also found by Rusli et al. (2006) with an improvement in homogenization pressure. Additionally, Rusli et al. (2006) found a marginal increase in microencapsulation effectiveness with an increase in homogenization pressure. The strong mechanical pressures that oil droplets and globular proteins experience during homogenization encourage oil droplet dispersion and protein structural alteration, like in expanding of protein chains (Rampon et al., 2003). Since polarized and nonpolarized protein regions are now accessible as a result of this unwinding, reactive amino acids have improved their surface activity. The biggest alterations to the amino acids' surface appearance may be seen in their secondary and tertiary structures. Since proteins are the emulsion's most surface‐active constituents, they build up at the oil–water contact and encircle the freshly created droplets of oil. This could be attributed to the fact that proteins tend to bind to hydrophobic surfaces more thoroughly and irreversibly than to hydrophilic ones. The resulting stabilizing layer gives the emulsion physical stability by immediately protecting the small droplets from recoalescence. Research has well established the fact that an increase in solid content improves the MEE, and that the emulsion's solid phase is a significant factor affecting active‐core stability (Charve & Reineccius, 2009). This could be explained by a high solid content, which also causes a reduction in the time required to form the protective shell and in the mobility of the core molecules in wall material. This may be described by the slower formation of the protective shell and decreased mobility of the core particles in wall material, both of which are caused by a high solid content. However, after passing almost 20% solid content concentration on weight basis passed, a rapid rise in viscosity is seen, which results in a considerable decline in procedure effectiveness (Yu et al., 2007). The drying intake temperature has been demonstrated to have an impact on the MEE from a process standpoint. In fact, an increased drying temperature promotes the development of a stiff surface shell of the wall component of the microparticle, restricting the mobility and release of the core molecules (Rascon et al., 2010). Efficiency of microencapsulation through spray‐drying is impacted by the volatile qualities of the core material; for example, MEE will be lower when encapsulating aromatic volatile cores as opposed to traditional oils. In fact, the high temperatures (150–180°C) that the liquid preparations are exposed to during the drying process cause evaporation of core, which accounts for the low efficacy of central microencapsulation by SPI (35% equated to stearin (91%) (Rusli et al., 2006)). The preservation of tocopherol, a hydrophobic active material during spray‐drying process of microencapsulation, can be enhanced as demonstrated in a recent work (Nesterenko et al., 2012) by grafting fatty acid chains (hydrophobic) to soy proteins through acylation. When dodecanoyl chloride was used to acylate soy proteins, process efficiency rose from 79.7% to 94.8%. Additionally, this improved retention effectiveness following acylation of proteins was shown for various wall/core fractions, proving that proteins found in soy, both in their natural and improved states, serve as an effective encapsulate agent for hydrophobic compounds (Lazko, Popineau, & Legrand, 2004). The coacervation method has also been used to study soy proteins as wall materials in microencapsulation, and it has been discovered that a number of characteristics affect the coacervation MEE. They include the concentrations of the active core and wall materials, the media's temperature, and its pH. Process efficiency typically declines as active hydrophobic core concentration rises beyond 50% weight to weight (Mendanha et al., 2009). The study of Mendanha et al. (2009) provides one of the best illustrations of this phenomenon, showing how a change in a component ratio between 1:1 and 1:3 resulted in a reduction in MEE from 92% to 79%. According to Jun‐xia et al. (2011), this propensity resulted from inadequate emulsification following the input of too much oil into the system. The electrostatic connections between gum arabic and soy proteins were impacted by nonemulsified oil, which led to emulsion instability. The stability and size of coacervates are directly correlated with the protein content (as wall material) during the emulsification process. This might be explained by the fact that oil droplet‐specific surfaces in emulsions are inversely related to their mean diameter (Lazko, Popineau, Renard, & Legrand, 2004). Because of the qualities of proteins as surfactants, raising protein concentration would enhance oil droplet‐specific surface, improve protein adsorption on the oil–water interface, and increase the droplets' resistance to coalescence, resulting in a reduction in mean diameter (generally detected by light scattering). However, Lazko, Popineau, Renard, and Legrand (2004) showed protein content did not appear to have a substantial impact based on the diameter of the microcapsule wall. According to certain writers (Lazko, Popineau, & Legrand, 2004), coacervation microencapsulation is more successful at high temperatures and acidic pH levels (55°C and 2, respectively). Denaturation of 11S globular proteins, which results in the transformation of their quaternary structure into tertiary and secondary structures, is favored in acidic media. This architectural modification can increase the total approachability of hydrophobic protein located inside the spherical forms (Nesterenko et al., 2013). Protein hydrophilicity declines as pH values fall below the isoelectric point and protein's COO‐ bond activities turn into uncharged carboxyl groups. The attraction between the active core (hydrophobic) and proteins in the emulsions would therefore be enhanced in an acidic medium, which should lead to better MEE. An interlinking phase is frequently inserted at the conclusion of the coacervation procedure when microencapsulation is being done, mostly to strengthen the microcapsule shells. The effectiveness of protein aggregation in the vicinity of oil droplets could not be affected by this additional step, but it would have a substantial influence on the emulsion's durability and, in turn, on the size and dispersion of the microcapsules, especially when stirring for a long time. Microcapsules coalesced without reticulation causing a noticeable rise in average microcapsule diameter (from 90 micrometer to more than 200 micrometer); however, this amalgamation was not present and no change in diameter of microcapsule got identified when microcapsules cross‐linked (Lazko, Popineau, & Legrand, 2004).Amajority cross‐linking substance is glutaraldehyde, which improves the mechanical characteristics and enables steady microcapsule dispersion over time (Lazko, Popineau, Renard, & Legrand, 2004). However, the relative toxicity of glutaraldehyde prevents it from being used in some industries, such as the food business (Chen et al., 2014). It was investigated how several hydrophobic hydrolysates were microencapsulated by spray‐dried soy protein was studied (Nesterenko, Alric, Silvestre, & Durrieu, 2014), and coacervation techniques (Mendanha et al., 2009) as to mask the bitter taste of hydrolysates having water‐phobic nature (e.g., casein hydrolysate) and then to mix these into edible products. The water‐phobic interfaces between soy proteins and casein hydrolysate during encapsulation reduce the casein's unpleasant flavor. The authors show that when active‐core concentration increases, microencapsulation effectiveness decreases and particle size increases. The creation of soy protein‐based microspheres using the cold gelation process (started in existence of calcium carbonate by glacial acetic acid) was described by Chen and Subirade (2009) to create sophisticated delivery systems for nutraceutical items (riboflavin). The attained microparticles were sphere shaped and had a 15 m diameter. Soy proteins effectively encapsulated active material at room temperature lacking the need for cross‐linking agents, which may be useful for a variety of culinary and pharmaceutical applications with process efficiency of around 79%–88%. Using both spray‐drying and coacervation procedures, multiple investigations have demonstrated the effectiveness of SPI as an encapsulating agent. However, the authors demonstrated that elevated level of MEE can be achieved in both situations through utilizing acceptable investigational settings. In both approaches, certain factors impact microparticle size and microencapsulation competence, notably the concentration of active core (Yu et al., 2007). The microparticles structure, size, and, consequently, the release of the bioactive material are typically greater with coacervation, and basically, are the main differences between the spray‐drying and coacervation.

### 4.2 Pea proteins

Without chemical or enzymatic alteration, polysaccharide/protein interactions do in fact provide pea proteins new activities, notably those related to foaming, surfactant, and most importantly, solubility characteristics (Liu et al., 2010). These interfaces lead to emulsions' stability, which can increase the microencapsulation process' effectiveness by resulting in improved particle size distribution. Pea proteins have been the subject of several investigations employing the spray‐drying method as the wall material for microencapsulation; the primary characteristics of the obtained microparticles. Combining the unique qualities of each of these polymers is possible when protein/polysaccharide combinations are used. Extracting pea proteins from pea seeds yields a 20%–30% fraction that is mostly composed of globulins (65%–80%) and two minor fractions, glutelins, and albumins. Legumin, vicilin, and convicilin are the three distinct proteins that makeup globulins (Adebiyi & Aluko, 2011). Convicilin and vicilin epitomize the 7S fraction of globulin having around 150 kDa molar mass, and pea legumin signifies the 11S fraction of globulin containing between 350 and 400 kDa molar mass. Pea proteins have intriguing gelling and emulsifying capabilities (Raymundo et al., 2005). However, these proteins are typically linked to polysaccharides in the literature for usage in microencapsulation (Ducel et al., 2004). When a surface‐active ingredient is present, polysaccharide products are typically employed as wall materials since they have lower emulsification characteristics than proteins. Incorporating carbs and proteins as encapsulating substrates increases durability of emulsion and provides improved antioxidant defense for active compounds (Liu et al., 2010). According to Gharsallaoui et al. (2010), proteins perform as an emulsifying and film‐forming agent in protein/carbohydrate blends, whereas polysaccharides serve as a material for constructing a matrix. Pea protein microparticles exhibit active‐core retention that is comparable to (or superior to) that of particles containing a protein/carbohydrate combination. This is most likely brought on by electrostatic interactions between proteins and the substance that is enclosed (Gharsallaoui et al., 2007). According to the literature, adding maltodextrin to the protein powder structural component enlarges the particles, especially when a hydrosoluble‐active ingredient is included (Pierucci et al., 2006). The notion that maltodextrin might hasten the production of a glassy surface, which would permit air expansion inside microparticles and result in an increase in particle diameter, was cited by the authors as justification for this rise. The findings show that pea proteins, either by themselves or in combination with polysaccharides, are perfectly suitable for microencapsulating hydrophilic (ascorbic acid) (Pereira et al., 2009). The utilization of globulin derived from peas (isoelectric point in a range of pH from 4.4 to 4.6) for the microencapsulation of triglycerides by complicated coacervation as well as the impact of pH and polymer concentration on the microcapsule size were all investigated by Ducel et al. (2004). The original formulation produced larger microcapsules when the concentration of the gum arabic/pea globulin (50:50) mix was increased. For instance, microcapsule sizes ranged from 28 m to 97 m at pH 3.5, with corresponding concentration changes of 1 g/L–10 g/L. Lazko, Popineau, and Legrand (2004) found that soy protein content increase was accompanied by a decrease in coacervate size. In actuality, the resulting microparticles' mean diameter shrank from 153 m to 88 m when the protein content rose from 0.5 g/L to 5 g/L, correspondingly. Differences in the coacervation procedure are likely what caused the discrepancy between the reported results. For pea proteins, complex coacervation was employed, and particle agglomeration and coalescence resulted in an increase in size. Coacervate agglomeration can be impacted by the original preparation's carbohydrate content (Klassen & Nickerson, 2012). While making soy protein microparticles, straightforward coacervation was employed. The emulsion's coalescence‐resistant coacervates increased as surface‐active protein concentrations rose. Additionally, the two coacervation procedures were not carried out under exactly the same circumstances. For instance, the soy and pea proteins were coacervated at 30°C and 55°C, with pH values of 2.0 and 3.5, and powered stirring at 500 rpm and 600 rpm, respectively. These investigations involved the creation of microparticles (with legumin or vicilin) using straightforward coacervation, and they appear to demonstrate that it is also feasible to coat an active substance without the inclusion of polysaccharides. In terms of the distribution of particle sizes, the range of the average particle size was 200–700 nm. In conclusion, whereas spray‐dried microspheres' typical diameters for microcapsules made from pea proteins ranged from roughly 10 to hundreds of microns, the normal size is never more than or equal to 10 μm (Table 1).

**TABLE 1:** Application of legume proteins for microencapsulation of different bioactive components

Method of encapsulation	Core component	Ratio from encapsulation to core	Type of legume	References
Freezing and homogenization	Oil from flaxseed	8.43:1 and 1.20:1	Chickpea	Gan et al., 2008
Utilizing high pressure to homogenize 2‐pass, 35–45 MPa, including freeze‐drying	Oil from fish	1:0.67	Soybean	Tang and Li (2013)
Spray‐drying and homogenizing	Ascorbic acid	1:2	Pea	Pierucci et al. (2006)
Complicated coacervation	Miglyol 812 N (MCT)	2.3:1	Pea	Gharibzahedi and Smith (2020)
Evaporation and homogenization 70 MPa, 1 cycle, including evaporation at 40oC	Beta‐carotene	1:1	Soybean	Tang and Li (2013)
Homogenization 5 rounds at 1 t 0.5 MPa	Olive oil	10–3:1: 0.1	Lentil	Tang and Li (2013)

## 5 ENCAPSULATING SYSTEMS USING PEA PROTEINS AS AN ADDITIVE

Pea proteins have emulsifying qualities that develop them possibly expedient as an addition to enhance emulsion stability rather than as a straightforward primary wall material. In order to create an oil‐in‐water emulsion with tiny oil droplets, Gharsallaoui et al. (2010) employed a little quantity of pea protein (0.5% w/w) as an emulsifier. Maltodextrin and pectin were then added creating an emulsion with triglyceride droplets covered in membranes of protein polysaccharide. This work supported the notion that fusing the traits of proteins and polysaccharides may be useful. Compared to emulsions made just of proteins, those including polysaccharides appeared to be less susceptible to changes in pH, strong ionic concentrations, and high temperatures (Wang et al., 2016). Additionally, the proteins' hydrophobic polypeptides that are introduced to emulsions made of polysaccharides have a high adsorption capability at oil–water interactions (for instance, pea globulin at an acid pH), which helps steady emulsions (Gharsallaoui et al., 2010). Experiments demonstrate that tiny amounts of protein may protect emulsions versus temperature, pH, and recrystallization changes. In conclusion, pea‐derived proteins have useful encapsulating capabilities and are utilized to preserve active ingredients or stabilize emulsions. The method of microencapsulation, the circumstances of the manufacturing process, and the usage of additives such as polysaccharides all had an impact on the properties of the final microparticles.

### 5.1 Wheat and other cereal proteins

Gluten, a particular protein found in wheat, is a byproduct of the separation of starch from wheat flour. They are complex substances made up primarily of proteins and a trace amount of polysaccharides. Gliadin and glutenin are its two primary constituents. As a single‐chain polypeptide having a molecular mass range of 25–100 kDa, gliadin is soluble in neutral 70% ethanol and is held together by intramolecular disulfide links. With a molecular weight more than 105 kDa, glutenin is a water‐soluble constituent made up of gliadin‐like subunits that are maintained in large aggregates by intermolecular disulfide bonds (Wang et al., 2016). Grain protein can be used alone or in combination with polysaccharides to successfully encapsulate active‐core components in a number of ways. Two protein fractions—glutelin and hordein—makeup the barley proteins that Wang et al. (2016) examined. These two fractions exhibit superb film‐forming and emulsifying abilities. Prolamin zein a protein fraction obtained from corn is well‐known for its excellent filmogenic capabilities and is soluble in hydroalcoholic solutions. Vaseline oil was encapsulated by a gliadin/gum arabic wall in a study by Lopretti Correa et al. (2017) that focused on the protein content and the impact of pH on the microcapsule characteristics. These numbers are consistent with those seen in microcapsules made of soy and pea protein. Grain proteins for solvent evaporation microencapsulation are the subject of very few investigations. Linoleic acid was encapsulated in a gliadin matrix, according to Iwami et al. (1988), to increase its stability and digestion, notably for making bread. Proteins isolated from barley seeds were employed by Wang et al. (2011) as a cover material for microencapsulation of oil extracted from fish, while proteins isolated from wheat were used as a capsule material for microencapsulation using the method of solvent evaporation. The microparticles were prepared using the spray‐drying process with a 150°C inlet temperature. The researchers showed substantial oil content in the powder—around 50%—and encapsulation efficiencies of 97%–100%. Grain proteins work well for encapsulating active‐core materials employing a variety of ways, either by itself or in conjunction with polysaccharides. Two protein fractions—glutelin and hordein—makeup the barley proteins that Wang et al. (2016) examined. These two fractions exhibit superb film‐forming and emulsifying abilities. Prolamin zein, a protein fraction obtained from corn, is well‐known for its excellent filmogenic capabilities and is soluble in hydroalcoholic solutions. Vaseline oil was encapsulated by a gliadin/gum arabic wall in a study by Lopretti Correa et al. (2017), that focused on the protein content and the impact of pH on the microcapsule characteristics. The produced barley peptide tiny particles have a spherical form, a porous interior, and diameters between 1 and 5 m. This protein was effective at preventing fish oil from oxidizing during meal preparation. Andreani et al. (2020) studied the impact of a modest quantity of polyethylene oxide on the characteristics of wheat gluten microspheres for the precisely measured release of a prototypical medication (diltiazem). They showed that it was possible to create completely spherical porous microspheres with mean particle sizes between 10 and 20 m and encapsulation efficiencies ranging from 73% to 97%. They demonstrated how greatly the MEE was enhanced by adding 5% w/w of PEO to the gluten matrix. This is most likely caused by the larger porosity of PEO‐containing microparticles, which results in a bigger specified surface region that favors better integration of the active‐core material. According to the solvent composition, Joye et al. (2015) found substantial differences in microparticle diameter, indicating the importance of physicochemical interactions between proteins and solvents. This study examined the role of solvent type. By encapsulating (antimicrobial) lysozyme from chicken egg white with zein, and expending a supercritical antisolvent technique, heterogeneously sized microparticles with a 46.5% MEE were produced. The kinetics of the active material release indicated highly positive microparticle characteristics for usage in food preparation. Briefly stated, cereal proteins are pertinent biomaterials that may be used as a microencapsulation medium. They work effectively to microencapsulate water‐fearing and water‐loving substances both on their own and when combined with artificial polymers and/or polysaccharides. Joye et al. (2015) stated that cereal proteins are pertinent biomaterials that may be used as a microencapsulation matrix. They work effectively for the microencapsulation of hydrophilic and hydrophobic substances both on their own and when combined with polysaccharides or artificial polymers (Table 2).

**TABLE 2:** Proteins from cereals are encapsulated

Wall coating	Core components	Method of microencapsulation	References
Corn zein	Quercetin	Method of antisolvent condensation	Patel et al. (2012)
Protein from barley	Oil from fish	Spray‐drying	Wang et al. (2011)
Corn zein	Lysozyme	Process using a supercritical antisolvent	Zhong et al. (2009)
Arabic gum/α‐Gliadin	Vaseline oil	Complicated coacervation	Ducel et al. (2005)
Corn zein	Essential oils	Phase separation	Parris et al. (2005)

## 6 OTHER VEGETABLE PROTEINS THAT MIGHT BE BENEFICIAL IN ENCAPSULATION

**FIGURE 3:**
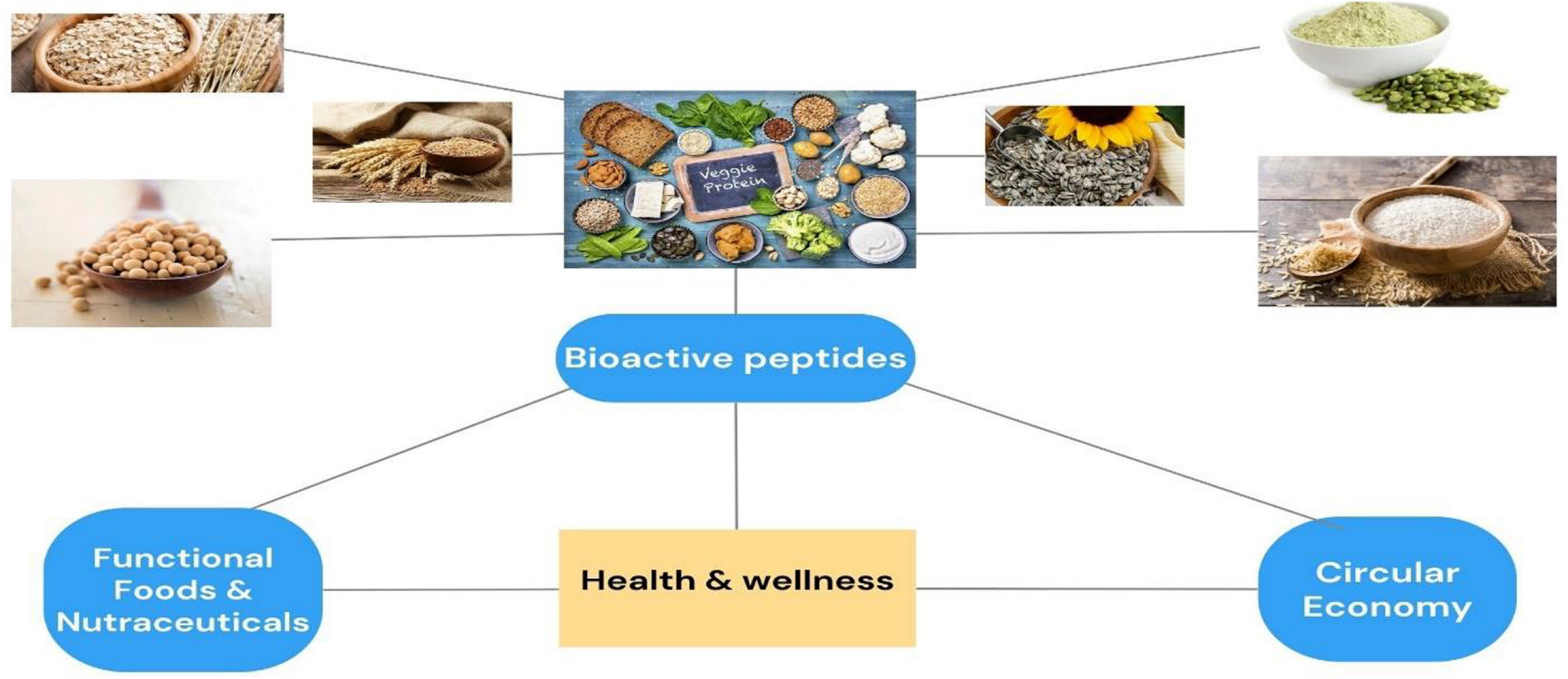
Flow sheet depicts the functional and nutraceutical benefits of vegetable proteins

Other proteins, particularly those found in rice, oats, and sunflowers, have qualities that make them potential candidates for use as wall material in microencapsulation. Already, the food industry uses rice and oat proteins for a wide variety of purposes. It might be exciting to discover new uses and create items with significant added value based on sunflower proteins despite the fact that their physicochemical properties have been thoroughly studied and that this natural polymer has few significant industrial usages **(**Figure 3).

### 6.1 Proteins from rice

One of the most significant cereal crops worldwide is rice. Moreover half of the globe's population relies on it as their primary source of nutrition. Rice bran, which is primarily taken from the grain during grinding methods to make white rice, contains between 12% and 20% proteins and might be a source of low‐cost, high‐quality proteins (Chandi & Sogi, 2007). The protein concentration of rice grains ranges from 6% to 15%, which is some what less than that of rice bran. In general, alkali extraction, isoelectric precipitation, and subcritical water treatment are used to make rice proteins. Rice has also been researched for its ability to produce starch, colors, and rice wine; as a result, rice protein may be another byproduct that may be used (Cao et al., 2009). Following the progressive extraction of the rice protein fractions, the resulting dissemination of glutenin, 15% globulin, 6% albumin, and 3% prolamin was discovered. The functional characteristics of rice protein concentrate were examined by Chandi & Sogi, 2007, which are 55% of the protein fraction. They observed the strong emulsifying capacity in sugar‐based solutions (5%–15% w/w) and the good emulsion stability depending on the sugar or salt content and pH. They also noted the outstanding foaming stability lasting several days. The physical and chemical characteristics are comparable to those of casein (Chandi & Sogi, 2007). Defatted rice bran is used to create rice bran isolate, which has been researched for its characteristics and has an approximate 92% protein content. Authors showed that rice protein has foaming abilities comparable to albumin from egg whites, which are better than those of rice protein; the maximum protein solubility is at pH 10; the minimum appears to be close to the isoelectric point at pH 4; the core amino acid amount of rice, casein, and soy proteins is similar; and the temperature of denaturation for rice protein isolate is approximately at 83.4°C. Additionally, rice proteins bind to carrageenan and alginate to generate complex precipitates that may have novel commercial uses (Fabian et al., 2010). These findings suggest that the physicochemical features of rice proteins may offer advantageous properties for wall matrix material during the procedure of microencapsulation. In contrast to possible low‐volume and high‐added‐value uses of microencapsulation, the utilization of rice protein often worries the food business.

### 6.2 Proteins from oat

Due to its high protein and fatty acid content, oats are among the most consumed cereals by both humans and animals. One of the largest sources of protein, oat grain contains between 12 and 24% of protein (Chronakis et al., 2004). Because oat proteins have a larger proportion of globulins and albumins as compared to other cereals, they have an exceptionally favorable average amino acid composition from the perspective of nutritional value. The majority of oat proteins come from globulin (around 70%–80%). Enzymatic hydrolysis, acetylation, and succinylation were used to modify these physicochemical characteristics, and it was shown that these chemical changes might improve oat protein solubility, foaming capability, and emulsifying activity (Guan et al., 2007). In conclusion, while oat natural proteins lack the qualities needed for microencapsulation, some particular changes could make it possible to employ them as wall materials.

### 6.3 Proteins from sunflowers

Sunflower seeds are one of the chief sources of edible oil and are primarily grown for the oil derived from its seeds. In sunflower seed cakes, which are primarily regarded as animal feed, proteins makeup the majority of the ingredients. Around 27% of the dry weight of the decorticated sunflower flour is made up of proteins (Ordonez et al., 2008). The amount of crude portentous matter in the dehulled seed ranged from 20% to 40% with the sunflower variety having a significant impact on this number (González‐Pérez & Vereijken, 2007). The amount of proteins recovered from sunflowers varies as well depending on the kind of solvent employed (most commonly water‐based solutions) and the extraction procedures (pH, stirring, and temperature). Four different protein fractions may be found in the sunflower oil cake: between 55% and 60% of the total are globulins, albumins makeup around 23% of all proteins, while glutelins and prolamins provide 11%–17% and 1%–4% of proteins, respectively (González‐Pérez & Vereijken, 2007). Sunflower proteins may be divided into two main fractions based on their sedimentation coefficients: the 11S globulins, also known as helianthins, and the 2S albumins. According to Patino et al. (2007), helianthin is a globular oligomeric protein that mostly appears in the 11S form and has a molecular weight of 300–350 kDa (hexametric structure). Helianthin can also take the 15‐18S, 7S, or 3S forms depending on the pH, ionic strength, temperature, and protein content. Different subunits of 11S sunflower proteins are conventionally processed to produce an acid as well as a basic polypeptide connected by a single disulfide bond. These polypeptides, which are both basic and acidic, have molecular weights that vary from approximately 21–27 kDa and about 32–44 kDa, correspondingly. Ionic strength and pH have a significant impact on how soluble helianthin is, with a pH minimum of 4–5.5. With molecular weights ranging from 10 to 18 kDa and a sedimentation coefficient of around 2S, sunflower albumin proteins exhibit property of being readily soluble in water‐based solutions, independent of ionic strength and pH. Conflicting the major fractions, there is no research describing the functional qualities of prolamins and glutelins from sunflower seeds. The majority of writers demonstrated that sunflower preparations had emulsifying qualities that are superior to those of soy protein preparations, or at least comparable. The main findings of these studies revealed that protein‐emulsifying capacity is not affected by the extraction method or solvent used for protein extraction and that heating that causes protein denaturation increases emulsion stability but decreases emulsifying capacities. The highest emulsifying capability is found in the pH range 7–8 and the lowest at the isoelectric pH of 4.3. The latter observation can be accounted for the modification of protein structure during denaturation due to heat, which favors unfolding chain and inclined flexibility in conformation. Because of this, unfolded sunflower proteins' surface‐active capacity decreases during emulsion formation, but it remains stable longer after emulsion preparation (Patino et al., 2007). Sunflower proteins appeared to form foam less effectively than soy proteins in terms of foam properties. Although stable over time at high concentrations and really basic pH level, sunflower protein foams. Sunflower proteins may undergo chemical alterations (for instance, enzymatic hydrolysis) that enhance their functional characteristics and result in novel, exciting uses (Conde & Patino, 2007). Sunflower proteins' potential as a source of human dietary proteins is restricted by the presence of phenolic chemicals, which give the powdered form of sunflowers its green–brown hue. Therefore, these proteins may have highly exciting new opportunities in industrial fields other than food. One potential industrial use for this agricultural byproduct would be microencapsulation.

## 7 APPLICATIONS OF NATURAL LEGUME PROTEINS (IN CONJUNCTION WITH POLYSACCHARIDES)

According to Nesterenko, Alric, Silvestre, & Durrieu, 2014; Nesterenko, Alric, Violleau, et al., 2014, polysaccharides can withstand a variety of processing conditions and are more soluble in water. Protein and polysaccharides together enhance the emulsification characteristics. Therefore, increased protein–carbohydrate combination qualities are a great strategy for efficient encapsulation. It has been discovered that the polysaccharide–protein combination demonstrated better stability than layer‐by‐layer biopolymer deposition (Gharsallaoui et al., 2012). Better stability was demonstrated by the polysaccharide‐like combination of protein and xanthan gum. By combining protein with gum, the continuous phase becomes viscous; also, a thicker network forms around the oil droplets, preventing the coalescence of the droplets. Abundant research has combined soy protein isolate with lupine. Comparing the emulsion stability of the pea protein and methyl pectin complex to that of the pea protein isolate alone in the face of creaming, the biopolymer (concentration, type, and size), emulsion formation techniques, and solvent conditions (salts, pH, and temperature) are the primary determinants of effective polysaccharide–protein interactions (Gumus et al., 2017).

## 8 PROBIOTIC ENCAPSULATION

Encapsulation is a useful technique for safeguarding probiotics throughout processing and storage. The controllable release mechanisms in encapsulation can transport probiotics to a specific target and release them at a certain moment (Afzaal et al., 2022). Probiotics are protected from stressful situations through encapsulation. Contrarily, during the preparation and storage of various meals, probiotic microorganisms that have not yet been encapsulated may be easily exposed to severe circumstances such as high atmospheric pressure, temperature, and osmotic pressure and low pH (Han & Baik, 2008). Acidic conditions in the stomach and bile in the intestinal tract may reduce the probiotics' chances of surviving (Afzaal et al., 2022). Microencapsulation is crucial for the survival of probiotics throughout storage and transit through the digestive system. Given their susceptibility to adverse environmental factors including air, temperature, moisture content, bile salt solution, stomach pH, etc., probiotics should be encapsulated. Microcapsule inclusion must not alter the sensory qualities of various meals (Charve & Reineccius, 2009). Microcapsules may provide an ideal anaerobic habitat for probiotic bacteria that are receptive. They protect against bacteriophages as well as adverse environments including cold and very acidic temperatures, lowering the likelihood of cell damage (Kailasapathy, 2006). Probiotic bacteria, such as Bifidobacterium and Lactobacillus, are one of the major types of living microorganisms that contribute to improved host health benefits in the gastrointestinal tract. Maintaining the probiotic's concentration around 106 and 107 CFU/g in any diet will allow it to operate therapeutically (Hugo et al., 2014). Numerous studies showed that soy protein isolate, a legume protein, is important for preserving probiotic stability and diversity in adverse circumstances. A protein structure comprised of native and modified isolates of soy protein, pea protein concentrates, and soy protein concentrates comprises *Lactobacillus delbrueckii* subspecies *lactis CIDCA 133* and *Lb. bulgaricus FTDC 1511* (*Lb. acidophilus*). According to Dianawati et al. (2017), *Lb. acidophilus* LA‐5's survivability ratio not only declines with higher storage temperatures (25–35°C) but microcapsule survival rates in simulated gastric fluid (SGF) are also shown to be less at higher temperatures. Similar results were seen for *Lb. bulgaricus FTDC 1511*, which showed lower viability post 21 days' storage at 25°C in contrast to 4°C. Additionally, compared to pea protein, soy protein isolate demonstrated marginally greater probiotic‐resistant encapsulant, according to the findings. According to Hugo et al. (2014), high‐tension soy protein isolates have the potential to preserve the sustainability of *Lb. delbrueckii subsp*. *Lactis CIDCA 133* over a period of 6 weeks while being stored at 4°C. SPI offers more protection during gastrointestinal transit due to its strong buffering capacity. Furthermore, the presence of sulfur‐rich amino acids cysteine and methionine in isolates of soy protein may squelch free radicals and O_2_ reactive species.

## 9 ENCAPSULATION BY VEGETABLE PROTEINS HAS INDUSTRIAL USES

The creation of emulsifiers, bioplastics, adhesives, and wall‐formulating materials for microencapsulation are only a few prospective uses for pea proteins that exhibit promising characteristics (De Graaf et al., 2001). However, it is not advised to utilize these proteins for technical purposes. Wheat proteins and maize zein's functional qualities also point to a number of possible uses for extractable polymers in the industries of biodegradable plastics, microencapsulation matrix materials, adhesives, cosmetics, and textiles. Both of these proteins have the potential to be useful candidates for microencapsulation, although there is still no real practical use for them. On the other hand, soya proteins have already been utilized in the food business as wall‐forming components, particularly to cover up the taste of specific nutritious additions (bioactive substances for athletes, like casein) (Ozkan et al., 2019).

## 10 FUTURE TRENDS

A large number of different types of matrices such as microparticle, nanoparticles, and hydrogels are manufactured with the help of food proteins. These matrices are prepared for different purposes in terms of functional or innovative food development. The properties of food proteins are important not only in determining the size of the product but also in terms of its texture, appearance, taste, and aroma; along with these benefits, they are also helpful in the determination of release rates of bioactive compounds as well as playing a role in measuring the dose that needs to be taken by the body to be bioavailable, and last but not least, the usefulness of composites. Although it helps in improving the techno functional aspects of leguminous proteins and each of the chemical and mechanical pretreatments was also used, there is a dire need for evidential research to conclude the parallel effects of high and low energy processes and enzymatic activity on the enhancement or progression of the functionality, constancy, and proteins' structure for the encapsulation of bioactive compounds. Previous studies had already proved that the role of nonthermal methods such as ultrasonication and high pressure would be helpful in improving functional properties of legume proteins (Zhao & Tang, 2016). Therefore, it is highly recommended in the synchronized use of ecoinnovative techniques such as cold plasma, high pressure, ultrasound, and pulsed electric field that are required for the encapsulation of probiotics and bioactive compounds to develop a denser protein network. By improving the manufacturing techniques along with fragile substance or nutraceutical stabilization strategies, these legume protein‐based substances will play important role in the improvement of functional food efficacy for future decades. Development in the technology field for making changes in the functional aspects of legume proteins would make these ingredients important wall materials for enhancing the probiotic viability and stability in functional foods (Afzaal et al., 2021).

## 11 CONCLUSION

Vegetable proteins are inexpensive protein sources to make edible coating for microencapsulation, especially to protect probiotics during transit from stomach juice and intestinal acids until they reach the targeted cell. Animal protein has been incorporated lately into microencapsulation but vegetable protein is more specific and economic than animal protein's action as microencapsulation material. Coacervation is a better process as compared to spray‐drying while contrasting particle properties during transit to unfavorable pH, temperature, and oxygen. There is a need to explore the kinetics and velocity rate of vegetable protein‐based microencapsulating materials alongside the safety until reaching the targeted site in the human body.

## FUNDING INFORMATION

The author has declared no conflict of interest.

## CONFLICT OF INTEREST

The authors have no relevant financial or nonfinancial interests to disclose.

## ETHICAL APPROVAL

This article does not involve humans or animals.

## CONSENT TO PARTICIPATE

All the coauthors are willing to participate in this manuscript.

## CONSENT FOR PUBLICATION

All authors are willing the publication of this manuscript.

## Data Availability

Even though adequate data has been given, however, all authors declare that if more data required then the data will be provided on request basis
